# Mass spectrometry as a tool for studying autism spectrum disorder

**DOI:** 10.1186/2049-9256-1-6

**Published:** 2013-05-21

**Authors:** Alisa G Woods, Armand G Ngounou Wetie, Izabela Sokolowska, Stefanie Russell, Jeanne P Ryan, Tanja Maria Michel, Johannes Thome, Costel C Darie

**Affiliations:** Biochemistry and Proteomics Group Department of Chemistry and Biomolecular Science, Clarkson University, 8 Clarkson Avenue, Potsdam, NY 13699-5810 USA; Department of Psychology, State University of New York at Plattsburgh, 101 Broad Street, Plattsburgh, NY 12901 USA; Department of Psychiatry, University of Rostock, Gehlsheimer Straße 20, D-18147 Rostock, Germany; College of Medicine, Swansea University, Singleton Park, Swansea, SA2 8PP UK

**Keywords:** Mass spectrometry, Proteomics, Protein biomarkers, Autism spectrum disorder

## Abstract

Autism spectrum disorders (ASDs) are increasing in incidence but have an incompletely understood etiology. Tools for uncovering clues to the cause of ASDs and means for diagnoses are valuable to the field. Mass Spectrometry (MS) has been a useful method for evaluating differences between individuals with ASDs versus matched controls. Different biological substances can be evaluated using MS, including urine, blood, saliva, and hair. This technique has been used to evaluate relatively unsupported hypotheses based on introduction of exogenous factors, such as opiate and heavy metal excretion theories of ASDs. MS has also been used to support disturbances in serotonin-related molecules, which have been more consistently observed in ASDs. Serotonergic system markers, markers for oxidative stress, cholesterol system disturbances, peptide hypo-phosphorylation and methylation have been measured using MS in ASDs, although further analyses with larger numbers of subjects are needed (as well as consideration of behavioral data). Refinements in MS and data analysis are ongoing, allowing for the possibility that future studies examining body fluids and specimens from ASD subjects could continue to yield novel insights. This review summarizes MS investigations that have been conducted to study ASD to date and provides insight into future promising applications for this technique, with focus on proteomic studies.

## Review

### Introduction

Mass spectrometry (MS) has been employed as a tool for understanding various biomedical disorders, and is more recently being used to investigate psychiatric disorders, including autism spectrum disorders (ASDs). Although initial MS studies have focused on identifying exogenous factors, more recent investigations are being conducted to understand endogenous protein changes and to identify possible ASD biomarkers. Here we summarize how MS has been employed to understand ASDs, and we provide a perspective on the likely future of investigations of ASDs using this technique.

Autism spectrum disorders (ASDs) are characterized by social deficits, repetitive/stereotypical behaviors and interests, as well as communication problems [1]. These disorders include autism, Asperger’s syndrome and pervasive developmental disorder not otherwise specified (PDD-NOS) [2]. Most recent available estimates of ASD indicate a prevalence of 1 in 88 US children. ASDs are more frequent in boys than girls, with a relative prevalence of one in 54 boys and one in 252 girls [3]. This indicates a continued increase in ASD incidence, however, the contribution of a true increase versus improvements in awareness and diagnosis is currently unknown [3]. Many researchers believe that improved detection alone does not account for the increased ASD incidence [4].

Current classifications for ASDs may change in the upcoming DSM-V (Diagnostic and Statistical Manual of Mental Disorders, fifth edition), in which autism will no longer be a separate diagnostic category within the larger classification of Pervasive Developmental Disorder, but rather encompassed under the broader ASD category. ASD will also encompass several syndromes that were individually classified in the DSM-IV-TR (DSM, fourth edition, Text Revision); Asperger's disorder and PDD-NOS will be eliminated [5]. Reflecting the common characteristics of ASD subtypes, diagnosis of ASD in DSM-V will require both “social/communication deficits” and “fixated interests and repetitive behaviors” to be satisfied [5]. It has been suggested that based on these new criteria, fewer individuals will be diagnosed with ASD [6]. This underscores the need for diagnostic means other than by simply measuring ASD symptoms.

It has been suggested that a small percentage of children diagnosed with ASD will spontaneously completely recover from symptoms, somewhere between 5-32% depending on the report [7]. However, these assessments are somewhat contradicted by the fact that ASDs are pervasive developmental disorders and therefore the diagnosis cannot be made if the symptoms are transient in nature. The existence of spontenous recovery remains an area of controversy. Behavioral interventions for ASDs, particularly when initiated at as early an age as possible, have been demonstrated to reduce ASD symptoms in 47-48% of cases [8–10]. Improvements in treatment are therefore still greatly needed. Despite the success of behavioral treatments, they are often initiated late due to delays in diagnosis. Pharmacological treatments of approved medications have not yet been demonstrated to be effective for ASD symptoms [11–13]. Several mGluR5 antagonist candidates as well as GABAergic agonists are currently being investigated for fragile X syndrome, the most validated and extensively studied genetic cause of ASDs [14]. These compounds may represent the first indicated medications for a known syndrome associated with ASD symptoms. There is no current medication indicated for ASD treatment, although individuals with ASDs are sometimes prescribed medication for various behavioral symptoms. They also may receive psychiatric medication for psychiatric comorbidities, such as depression. ASDs often are accompanied by other psychiatric problems [15, 16].

Current theories of ASD etiology point toward a genetic and environmental interaction [17]. A large body of evidence on genetic information in ASDs currently exists [18–21], although this has not made the etiology of the disorder any less equivocal. Based on a twin study, risk for ASD is more than 20-times increased in first-degree relatives [22]. Other studies have reported from 60% to 92% ASD concordance in monozygotic twins and from 0% to 10% in dizygotic twins [22, 23]. Despite this evidence for a strong genetic component, conclusively associated genes have remained unidentified, with over 100 genes and 40 genomic *loci* reported as being associated with ASD [24, 25]. A recent study of *de novo* mutations in ASD indicates that many mutations found in individuals with ASDs are in fact unassociated with the disorder [26]. This evidence points toward possible heterogeneity of ASD genetic causality [27] and also indicates that genetic information may not be sufficient to understand ASD etiology. Multiple genes in conjunction with environmental factors may interact to cause ASD [27–29]. The complexity of ASD makes genetic testing for ASDs relatively unsuccessful in many instances [28]. Although numerous studies have pointed to various biological disturbances present in children with ASDs [30–33], no current biological marker is available to aid in ASD diagnosis. ASDs are diagnosed based on behavioral symptoms, including language delays. ASDs therefore often go unrecognized until a child is at least 2 years old or even older, and current screening instruments for ASDs generate many false positives [34].

**Figure 1:**
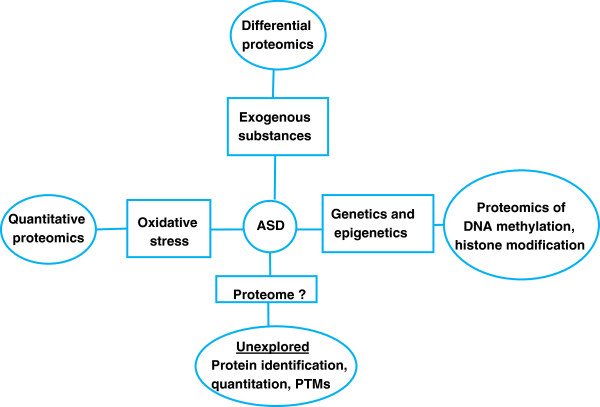
**Current proteomic approaches for MS investigations in ASD.** Several studies have suggested the causality of ASD to be of genetic and/or epigenetic origin as well as to involve exogenous toxicants (metals, environmental contaminants. MS studies have therefore initially focused on identification of exogenous substances. The proteome, which constitutes the most dynamic entity in human physiology, has not been as extensively studied. Mass spectrometry can also be used for the analysis of the proteome of ASD samples. This includes modifications of DNA, such as methylation or histone modification, qualitative proteomics, which identify protein patterns in controls versus ASD without necessarily implicating specific proteins and ASD proteome identification. This last area of study may be particularly promising for shedding light on ASD etiology or providing potential diagnostic biomarkers.

In this regard, we advocate the use of the mass spectrometer as a possible screening instrument for ASDs for many reasons. High-performing mass spectrometers, simplified analytical workflows and versatile data analysis tools render the technique easy, fast, accurate, reproducible and reliable compared to genetic studies. Further, dynamics at the environmental, developmental and genetic stages are truly reflected at the protein level. Moreover, proteomics could lead to the discovery of proteome fingerprints or profiles specific to ASD and therefore to biomarker discovery for the diagnosis, prognosis, monitoring and target identification for treatments for this disorder (Figure 1).

### Mass spectrometry and proteomic analysis in autism spectrum disorders

**Figure 2:**
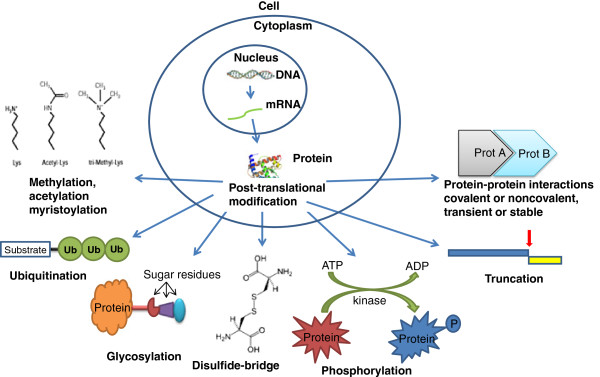
**Most common protein PTMs possibly relevant in ASD.** Despite the numerous susceptibility genes advanced for ASD, none of these genes has been confirmed to date. Perhaps, genetic disturbances in ASD manifest themselves first on the protein level as aberrant modifications of a protein or a set of proteins (e.g. truncation, phosphorylation, glycosylation, disulfide-bridge or protein-protein interaction).

Proteomics has the power to complement genetic information by identifying subtypes of ASD, or by identifying common protein dysregulations caused by different genes [35]. Proteomics is the study of the total complement of proteins at the time of harvest in a specific cell, organelle, extracellular fluid or tissue [36]. Proteomics can convey information that goes beyond genomic analysis, such as indications of active protein levels [37] or possible important post-translational modifications such as glycosylation, phosphorylation, and formation/destruction of disulfide bridges that maintain or disturb the protein’s three-dimensionality [35], summarized in Figure 2. The mass spectrometer is the workhorse of proteomic analyses but it can also be used to identify other relevant compounds [38] potentially associated with ASDs, such as environmental or ingested toxins or metabolites [39].

**Figure 3:**
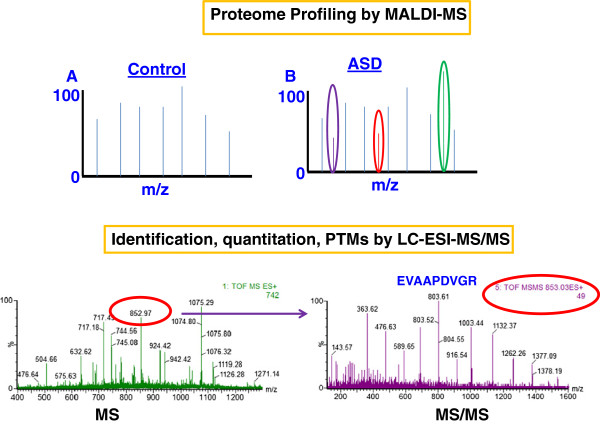
**Both MALDI**-**MS and LC**-**ESI**-**MS**/**MS can be considered for the study of the proteome of ASD samples.** MALDI-MS is more suitable for profiling studies, whereas LC-ESI-MS/MS allows for an in-depth data analysis for protein identification and quantitation as well as for the search of post-translational modifications.

Several different bodily fluids or products can be analyzed by MS: urine, blood sera, saliva, hair and tissue biopsies (Figure 3). Proteomic analysis of brain tissue, including synaptic proteins, using MS is also possible, although this approach tends to be limited to ASD animal models [40, 41]. There are different reasons why a specific bodily fluid or specimen is chosen for a particular proteomic experiment. Blood serum contains proteins, nucleic acids, lipids, and other metabolic products due to the nature of blood as a complex liquid tissue. This makes blood serum a rich source of disease biomarkers [42]. In contrast to blood, urine can be obtained non-invasively and in large proportions. Urinary proteins consist mainly of plasma proteins and proteins secreted from the kidney and urinary tract. The lower complexity of the urinary proteome compared to the serum proteome makes detection of low-abundant molecules much easier. Saliva is comparable to urine with the added benefit that it contains less salt than urine. Salts are not desired in MS experiments [43].

MS analysis of proteins or peptides mixtures allows the identification of proteins by measuring the mass-to-charge ratio of gas phase ions. A mass spectrometer is made of three main parts: an ion source, an analyzer, and a detector. The ion source is the part of the mass spectrometer where molecules are converted into gas phase ions which are then separated in the analyzer and the signal is generated by the detector. The most common ion sources are MALDI (Matrix-Assisted Laser Desorption/Ionization) and ESI (ElectroSpray Ionization). Ionization in a MALDI source occurs through a pulsed laser beam that sublimates molecules whereas in ESI, ionization takes place by subjecting liquid droplets to high voltage at atmospheric pressure [44]. There are several mass analyzers: TOF (Time-Of-Flight), quadrupole, ion trap, and FT-ICR (Fourier Transform Ion Cyclotron Resonance) [45]. There are variants of MS, in which the intact proteins are analyzed (top-down proteomics), or the protein molecules are first digested (e.g. by Trypsin) and the resulting peptides are then analyzed by MS (bottom-up proteomics) [36, 46–60]. Of the two approaches, bottom-up proteomics is the most established because of some of the limitations of the top-down approach; it is restricted to simple protein mixtures, instruments are very expensive and there are difficulties in analyzing high molecular weight proteins, the presence of post-translational modifications within the proteins, as well as the presence of both intact and truncated proteins within the same sample [61]. When the peptides generated in bottom-up approaches are first separated by liquid chromatography (LC) prior to MS, this approach is termed shotgun proteomics. The signal obtained from an MS experiment is given in the form of spectra, which are then compared to a database for protein identification. We will review and summarize the results obtained with different analyses of biological materials for the understanding of changes that occur in ASD versus controls.

### Urinary analysis in ASDs

Of the human biomaterials, urine has been most frequently studied in ASD research. For example, Cass et al., (2008) used high pressure liquid chromatography (HPLC) and Matrix-Assisted Laser Desorption/Ionization-Time-Of-Flight Mass Spectrometry (MALDI-TOF MS) to analyze the urine of 65 boys with autism, compared to the urine of 158 male controls of approximately the same age. MALDI-TOF MS is the best MS method for determining peptide or protein mass and for identifying a protein from a peptide mixture [62]. The purpose of this study was to test the hypothesis that exogenously-derived opioid peptides are found in the urine of children with autism, which has been one prevalent view of autism causality, albeit with little empirical support [63]. Indeed, using MALDI-TOF MS and HPLC, these investigators found no significant differences between the urinary profiles of children with autism versus controls, including an absence of differences in urinary opioid peptides [64]. In another study by Hunter et al., (2003), the same hypothesis was tested using LC-UV-MS (Liquid Chromatography-Ultraviolet-MS) and again the authors could not confirm previous findings that autism results from an opioid peptide excess [65].

A separate study examining the possible presence of opioids in subjects with ASDs used a highly sensitive Liquid Chromotography-ElectroSpray Ionization-tandem Mass Spectrometry (termed LC-ESI-MS/MS or LC-MS/MS for short). This approach allows a researcher to not only identify a protein, but also to determine the protein sequence [62]. MS/MS mode refers to a process by which the mass spectrometer records the peptides’ spectra (MS mode) and then peaks are selected according to predefined criteria (e.g. charge state, intensity) for fragmentation via collision-induced dissociation (CID) to produce product ions (MS/MS mode). Thus, this mode makes it also possible to select target peaks or peaks of interest for sequencing or quantitation (single reaction monitoring, SRM) [62]. The researchers developed this method to be selective for opioid peptides in the urine of 54 children with ASD versus 15 age-matched controls using a method called solid-phase extraction (SPE). The targeted peptides included gliadinomorphin, beta-casomorphin, deltorphin 1, and deltorphin 2. These investigators also found no evidence for the presence of excessive opioid peptides in the urine of children with ASDs relative to controls [4]. Based on these results and the previously mentioned studies, continued investigation of the opioid hypothesis does not seem to be warranted and clinical recommendations based on the opioid hypothesis would appear to be unfounded in scientific evidence.

Inductively Coupled Plasma-Mass Spectrometry (ICP-MS) was used to test a different relatively unsupported theory of autism—that children with autism excrete an excess of heavy metals [66, 67] and that metal chelators can serve as a treatment for autism. ICP-MS is appropriate for analyzing the elemental composition of materials and is therefore good for analyzing metals [68]. Unfortunately, this study tested few subjects; 15 children with autism and 4 controls completed the study. Arsenic, cadmium, lead and mercury urine content was tested during a “provoked excretion” versus baseline. Only three children excreted more metal than baseline during the provoked excretion; two near the level of detection. A third child excreted mercury, but this was corrected based on a dietary change. Overall, this study did not support a heavy metal toxicity theory of autism [69]. This is in contrast to a study examining the heavy metal content of hair. Kern and colleagues examined the presence of sulfhydryl-reactive metals (mercury, lead, arsenic, and cadmium) in the hair of 45 children with autism (1–6 yrs of age) versus 45 gender-, age-, and race-matched typical children. Arsenic, cadmium, and lead were significantly lower in the hair of children with autism as compared to matched controls. Mercury was also lower in the hair of children with autism, but not significantly [39]. The authors concluded that their results could indicate that children with autism have more difficulty in excreting toxic metals versus matched typical children [39]. The detection of heavy metals being low in the hair of children with ASD is an interesting finding however, this theory does not seem to be consistently supported by other studies examining the same heavy-metal excretion theory.

In addition to the measurement of exogenously-introduced factors, urinary analysis of endogenous factors, such as metabolites, is possible using MS. A study utilizing LC-MS/MS to analyze the urine of young adults with severe ASD and schizophrenia versus healthy controls (n=15, 18 and 18, respectively) found that butofenine, a molecule related to serotonin (a neurotransmitter that may be dysregulated in ASD) [70], was elevated in individuals with ASD and schizophrenia. Butofenine derives from the double-methylation of serotonin and may have hallucinogenic properties [71, 72]. Butofenine also correlated with scores of hyperactivity in these individuals [70]. Although the subject numbers were not high in this study, the results are consistent with a pattern of serotonin abnormalities that have been observed in numerous ASD studies [63]. Abnormalities in the serotonergic system were also observed in a study of 33 children with autism versus 21 healthy children, which used gas chromatography–mass spectrometry (GC-MS) to examine urinary levels of tryptophan (an amino acid responsible for bodily serotonin production). This form of MS is commonly used for metabolites in blood and urine, among other applications [73]. Urinary tryptophan was significantly lower in children with autism than healthy controls, including those on a restricted gluten- and casein-free diet [74]. An early study using HPLC and mass spectrometry found that increases in a serotonin-uptake peptide were also observed in the urine of 67% of children diagnosed with autism (n = 135) using DSM-III-R (DSM, third edition, Revised) criteria when compared to healthy controls (n = 126) [75].

MS evidence supports an oxidative stress theory of autism, another current prevalent theory [17]. Oxidative stress refers to the generation of reactive oxygen species which can cause cellular and tissue damage [62]. GC-MS was used to measure urinary levels of markers for lipid peroxidation (isoprostane F(2alpha)-VI), platelet activation (2,3-dinor-thromboxane B(2)) and endothelium activation (6-keto-prostaglandin F(1alpha) in children with autism (n = 26) versus healthy control subjects (n = 12). Subjects with autism had higher urinary markers for lipid peroxidation and endothelium activation. Levels of lipid peroxidation correlated with levels of platelet and endothelium activation [76]. MS is an effective means for measuring oxidative stress markers [62] and further mass spectrometric studies of ASD samples can help to clarify the role that oxidative stress may play in ASD.

Taken together, these MS analyses have supported that oxidative stress and dysregulation of serotonin and related molecules may be present in ASD. These studies did not however, strongly support theories involving the introduction of exogenous substances such as heavy metals and diet-derived opioids as causal factors in ASDs. Based on these MS findings and additional studies, the field has moved forward in the study of endogenous molecules in ASD using MS, and specifically proteomics.

### Blood sera analysis in ASDs

Blood serum is one of the most common bodily fluids that have been studied in ASD, primarily for examining endogenous changes. This is not always a feasible method, due to the invasive nature of a blood draw and the possible reluctance of children and their parents to allow this. Taurines et al., (2010) found protein differences in 16 subjects with ASD and ADHD (n = 9, without ADHD n = 7; ADHD = attention deficit hyperactivity disorder) versus 16 age-matched controls. They reported three peaks with differences, however did not aim to definitively identify the actual proteins [77]. They have suspected that the protein may be an apolipoprotein [38]. Corbett et al., (2007) found elevations in apolipoprotein (apo) B-100 and apo A-IV in children with high functioning versus low-functioning autism. These researchers also observed alterations (upregulation) in complement factor H-related protein, complement C1q and fibronectin 1 and apoB-100 in children with autism versus matched controls [78]. These results showing changes in apolipoproteins (responsible for cholesterol metabolism and transport) are consistent with current theories proposing that disturbances in the cholesterol system may underlie some forms of ASD [33, 79, 80].

Promising mass spectrometric analyses using RP-LC-ESI-TOF-MS or MALDI-TOF-MS (common types of MS instruments) [77, 78] of blood in subjects with ASD have begun to emerge from the Childhood Autism Risk from Genetics and the Environment (CHARGE) Study being conducted at the University of California, Davis, a large study addressing numerous susceptibility factors for autism, and their interactions [81]. For example, blood mercury levels were found to be unaltered relative to controls in children 2–5 years old with autism or ASD (with developmental delay) relative to controls [82] (n = 249, n = 60, n = 143, respectively). Possible presence of polybrominated diphenyl ethers (PBDEs) in children with autism and developmental delay were also assessed in 100 subjects from this study using GC-MS. No differences were observed in the plasma levels of PBDEs in children with autism or ASDs/developmental delay in this study [83]. This report again emphasizes the utility of MS methods for assessing the presence of possible environmental toxins in individuals with ASDs. It also underscores the need to assess subjects from different populations and geographical regions in order to examine the possible influence of environmental contaminants in the etiology of ASDs. Russo and Devito [84] found increased plasma levels of copper during analysis of plasma from 79 individuals with autism, 52 individuals with PDD-NOS, 21 individuals with Asperger's Syndrome using ICP-MS. Further, they investigated zinc plasma levels and found in this case no significant alterations in zinc concentrations in individuals with autism, PPD-NOS and Asperger’s Syndrome. Zinc and B-6 therapy resulted in a decrease in copper concentration in autism and PPD-NOS patients but not in patients with Asperger’s Syndrome whereas the zinc levels were higher in all of the three conditions [84].

An addition application for MALDI-TOF in the study of ASD lies in the analysis of genomic modification, such as DNA methylation. For example, MALDI-TOF-MS has been shown to be more suitable for high-throughput methylation analysis in blood testing of individuals for the fragile X syndrome than the current gold-standard for molecular diagnosis of this disorder, Southern blotting. One study showed that differential methylation of two fragile X-related epigenetic elements within the fragile X mental retardation gene intron 1 (FMR1) was related to depletion of fragile X mental retardation gene product (FMRP) expression [85]. An additional study examining FMR1 intron 1 methylation used MALDI-TOF MS to analyze the blood of 74 controls compared with 62 premutation (between 55–200 CGG repeats in the FMR1 gene) or 18 full mutation (more than 200 CGG repeats) females. These investigators found that FMR1 intron 1 hyper-methylation was predictive of verbal cognitive impairment assessed using Wechsler intelligence quotient tests [86]. This is an example of how MS can be used to measure a biochemical parameter that is predictive of behavior. As other ASD biomarkers are revealed, these may also be correlated with behavior and used to predict ASD severity. Such information could in turn be used to guide types of treatments and when to initiate treatment.

### Salivary analysis in ASDs

Saliva has been assessed using mass spectrometry and saliva components match the contents of blood in many instances [87–89]. This is also a less invasive method for examining protein differences in individuals with ASDs. Saliva may provide an accessible biomaterial identifying biomarkers, for example, elevated phosphorylated tau has been measured in the saliva of subjects with Alzheimer’s disease relative to controls [90] and salivary uric acid has been used as a biomarker for metabolic syndrome [91]. However, only one published study examining saliva components in ASDs is currently available. Castagnola and colleagues (2006) used one type of MS (HPLC-ESI-Ion Trap-MS or High Performance Liquid Chromatography- ElectroSpray Ionization- Ion Trap- Mass Spectrometry) to measure salivary peptides in individuals with ASDs (12 with an autism diagnosis, 1 with Asperger’s Syndrome, 14 with PDD-NOS) versus age-matched controls. Phosphorylation levels of statherin, histatin 1 and acidic proline-rich proteins (entire and truncated isoforms) were significantly lower in subjects with ASDs. Lower phosphorylation levels of at least one peptide was found in 66% of all ASD subjects [92].

### Limitations of MS

MS is a versatile tool that one can use in investigation of bodily fluids such as sera, saliva or urine (and many other fluids). However, when MS is used to investigate these fluids, several factors have to be kept in mind, such as sample preparation, sample investigation, data analysis and data interpretation. Each of these steps in which the sample is being processed also has some limitations and one should be aware of them. Perhaps one of the greatest features of a MS-based experiment is both an advantage and a limitation One MS experiment can produce an enormous amount of information/data, however, limitations of MS experiments lie within the data analysis. Although one can obtain a high amount of data, there is a challenge is in interpreting the results. In the most successful MS experiments we do not know or understand the meaning of about 30% of the spectra. Therefore, no matter how many weaknesses one MS instrument has (e.g. dynamic range, resolution, etc.), the bottleneck and limitation of a MS experiment is restricted to data interpretation, or bioinformatics. Due to the large amounts of data and need for interpretation, one may focus on one set of proteins selecting a set with both biological and clinical relevance. Therefore, the limitations of the MS-based experiments are not MS instrument-based, but rather bioinformatics-based limitations.

## Conclusions

Proteomics and investigation of possible ASD gene products is one clear application for MS in ASDs. The proteomic researcher using MS needs to consider however, refinements of MS techniques for protein analysis. Simply measuring proteins levels and looking for changes are not sufficient, interpretation of data is also important, if not the key. A proteomics experiment using MS involves several steps, and these steps are continuing to be refined by researchers. Specifically: 1) sample fractionation, enzymatic digestion of proteins into peptides, 3) analysis, 4) database search and interpretation, and 5) data validation, typically using Western blotting (WB) [36]. For example, our recent study found that peptides containing a cysteine residue will be identified as alkylated, but also other peptides. The akylation of non-cysteine-containing peptides was not previously possible to consider in a proteomic database search. This emphasizes that database searching also is in need of refinement in order to improve MS analysis. Therefore, we proposed that during a protein search the researcher should have the option of searching for any alkylated peptide at the N-terminal amino acid [59].

In addition to continued refinements of MS techniques, proteomic studies using MS tend to focus on identification of novel proteins [57, 60, 93, 94] or on increases and decreases in proteins [54, 58], but there are other aspects of protein modifications that can be studied. For example, structural characterization, post-translational modifications or alterations in both stable and transient protein-protein interactions may all be a subject of study [33, 36, 46–48, 55–57, 59, 62, 93–97]. These changes can modify protein function and are important to consider. For example, have recently described the combination of Blue Native PAGE (BN-PAGE) or Colorless Native PAGE (CN-PAGE) with MS as a powerful means for measuring protein-protein interactions [36, 48, 49, 52, 95, 97]. Additional refinements in proteomic analysis using mass spectrometry continue to be developed [48, 53–57, 93, 94, 96, 97] and will undoubtably increase the already formidable power of this technique. Proteomic analysis can also be accomplished at several levels, including supracellular, subcellular, intracellular, extracellular, peptides, proteins or protein complexes. Proteomic analysis can focus on classical structural or functional analysis and also on a specific aspect of the proteome, such as protein oxidation, phosphoproteomics, protein methylation or protein secretion [53, 62, 85, 86, 92, 98].

### Future directions

Several putative markers for ASDs have been identified using mass spectrometry applied to various human biomaterials. Such markers include serotonergic markers, indications of oxidative stress, hypophosphorylation, hypermethylation (in the case of Fragile X) as well as apolipoprotein and complement protein dysregulation. Future studies may concentrate on additional post-translational modifications, such as protein nitrosylation, carbonylation, eliminylation. These are modifications that occur in human conditions and because they denature a protein’s function most of the time irreversibly they may be of interest for study in ASD.

The numerous different markers found in ASD samples may be reflective of multiple elements that are disturbed in ASDs and may also reflect ASD heterogeneity. This heterogeneity is also mirrored in the more than 100 genes and 40 genomic *loci* brought in association with ASD. This implicates that large pools of samples are needed in order to produce results with statistical power. Current theories propose that ASDs have multiple subtypes and etiologies. Future proteomic studies, including those utilizing mass spectrometry, may therefore reveal a biomarker signature unique to each subtype. Biomarker signatures may in turn aid in subtyping ASDs or in determining that different gene mutations may result in common protein perturbations. Correlation of mass spectrometric protein evidence with behavioral measures could additionally aid in defining protein biomarkers as predictive indicators of ASD behavioral symptoms, indeed at least one study has confirmed this possibility [86]. Refinements of protein analytical techniques, such as the development of more sensitive instruments with the ability to detect more proteins, complemented with advanced bioinformatics software for data interpretation will undoubtably advance the field. The availability of new technology will make the re-analysis of biological products in subjects with ASD versus matched controls possible and worthwhile and with the potential to yield additional novel results.

## Abbreviations

ASD: Autism spectrum disorders
PDD-NOS: Pervasive Developmental Disorder Not Otherwise Specified
DSM-V: Diagnostic and Statistical Manual of Mental Disorders, fifth edition
DSM-IV-TR: DSM, fourth edition, Text Revision
CHARGE Study: Childhood Autism Risk from Genetics and the Environment Study
PBDEs: Possible Presence of Polybrominated Diphenyl Ethers
FMR1: Fragile X Mental Retardation Gene Intron 1
FMRP: Fragile X Mental Retardation Gene Product
MS: Mass Spectrometry
MALDI: Matrix-Assisted Laser Desorption/Ionization
ESI: ElectroSpray Ionization
TOF: Time-Of-Flight
FT-ICR: Fourier Transform Ion Cyclotron Resonance
LC: liquid chromatography
LC-UV-MS: Liquid Chromatography-Ultraviolet-MS
LC-ESI-MS/MS or LC-MS/MS: Liquid Chromotography-ElectroSpray Ionization-tandem MS
SRM: Single Reaction Monitoring
SPE: Solid-Phase Extraction
ICP-MS: Inductively Coupled Plasma-Mass Spectrometry
WB: Western blotting.
